# First detection of *Cryptosporidium* spp. in red-bellied tree squirrels (*Callosciurus erythraeus*) in China

**DOI:** 10.1051/parasite/2019029

**Published:** 2019-05-13

**Authors:** Yijun Chai, Lei Deng, Haifeng Liu, Jingxin Yao, Zhijun Zhong, Leiqiong Xiang, Hualin Fu, Liuhong Shen, Ziyao Zhou, Junliang Deng, Yanchun Hu, Guangneng Peng

**Affiliations:** The Key Laboratory of Animal Disease and Human Health of Sichuan Province, College of Veterinary Medicine, Sichuan Agricultural University Chengdu Sichuan 611130 PR China

**Keywords:** *Cryptosporidium*, Zoonosis, Red-bellied tree squirrel, *Callosciurus erythraeus*, SSU rRNA

## Abstract

*Cryptosporidium* spp. are opportunistic pathogens that cause diarrhea in a variety of animal hosts. Although they have been reported in many animals, no information has been published on the occurrence of *Cryptosporidium* spp. in red-bellied tree squirrels (*Callosciurus erythraeus*). A total of 287 fecal specimens were collected from Sichuan province in China; the prevalence of *Cryptosporidium* spp*.*, measured by nested-PCR amplification of the partial small-subunit (SSU) rRNA gene, was 1.4% (4/287). Three different *Cryptosporidium* species or genotypes were identified: *Cryptosporidium parvum* (*n* = 1), *Cryptosporidium wrairi* (*n* = 1), and *Cryptosporidium* rat genotype II (*n* = 2). The present study is the first report of *Cryptosporidium* infection in red-bellied tree squirrels in China. Although there is a relatively low occurrence of *Cryptosporidium*, the presence of *C. parvum* and *C. wrairi*, which were previously reported in humans, indicates that red-bellied tree squirrels may be a source of zoonotic cryptosporidiosis in China.

## Abstract (fr)

Les *Cryptosporidium* spp. sont des agents pathogènes opportunistes qui provoquent une diarrhée chez divers animaux hôtes. Bien qu’ils aient été signalés chez de nombreux animaux, aucune information n’a été publiée sur la présence de *Cryptosporidium* spp. chez les écureuils à ventre rouge (*Callosciurus erythraeus*). Au total, 287 spécimens fécaux ont été recueillis dans la province du Sichuan en Chine. La prévalence de *Cryptosporidium* spp., mesurée par amplification par PCR imbriquée du gène partiel de la petite sous-unité de l’ARNr (SSU), était de 1,4 % (4/287). Trois espèces ou génotypes différents de *Cryptosporidium* ont été identifiés : *Cryptosporidium parvum* (*n* = 1), *Cryptosporidium wrairi* (*n* = 1) et le génotype II de *Cryptosporidium* du rat (*n* = 2). La présente étude est le premier signalement d’infection à *Cryptosporidium* chez des écureuils à ventre rouge en Chine. *Cryptosporidium* est relativement peu présent, mais la présence de *C. parvum* et de *C. wrairi*, déjà signalés chez l’homme, indique que les écureuils à ventre rouge peuvent être une source de cryptosporidiose zoonotique en Chine.

## Introduction

*Cryptosporidium* spp. are causative agents of cryptosporidiosis and common intracellular parasites that can infect a wide range of vertebrates, including humans [4]. Humans and other animals are mainly infected via the fecal–oral route, directly from infected humans or animals or indirectly from food or water contaminated by feces of infected hosts [4, 28]. In humans, *Cryptosporidium* infection can be asymptomatic or cause mild symptoms in immunocompetent individuals; however, in infants, young animals, and immunocompromized individuals, it may cause fatal and chronic diarrhea [3, 6, 7].

Based on molecular epidemiologic surveys of cryptosporidiosis in different host species, at least 31 valid species and more than 70 genotypes are recognized, with new genotypes continually being found [9]. Among them, the most common causative agents for cryptosporidiosis are *C. hominis*, *C. parvum*, *C. ubiquitum*, and *C. meleagridis*, though nearly 20 *Cryptosporidium* species and genotypes have been reported in humans [11, 25]. Some species of *Cryptosporidium* are host-adapted and have a narrow host range, such as *C. muris*, *C. andersoni*, and *C. suis*, mainly infecting rodents, cattle, and pigs, respectively, whereas *C. parvum* and *C. ubiquitum* have been identified in various animals [2, 18].

In China, *Cryptosporidium* spp. have been reported in humans, animals, and water samples; however, only limited reports on pet squirrels are available, and their role as reservoirs of infection for humans and other animals is unknown [10]. Red-bellied tree squirrels (*Callosciurus erythraeus*) are common companion animals closely associated with humans, and a previous study identified that they can harbor the human pathogen *Enterocytozoon bieneusi* [8]. Therefore, the main objective of this study was to determine the occurrence and the species/genotypes of *Cryptosporidium* in red-bellied tree squirrels and to assess their public health significance.

## Materials and methods

### Ethics

The present study protocol was reviewed and approved by the Research Ethics Committee and the Animal Ethics Committee of Sichuan Agricultural University. Permission was obtained from the owners or shop managers before the fecal specimens were collected.

### Sample collection

Two hundred eighty-seven fresh fecal specimens from red-bellied tree squirrels were collected from four pet markets between March 2017 and September 2018 in Sichuan province, China. Each red-bellied tree squirrel was kept in a separate cage in four pet markets. Approximately 30–50 g fresh fecal samples were collected from the bottom of each cage after defecation using sterile disposal latex gloves and then immediately placed into individual disposable plastic tubes. The fecal samples were transported to the laboratory with ice packs within 24 h of collection. No obvious clinical signs were observed during the sampling process. All fecal samples were stored in 2.5% potassium dichromate at 4 °C prior to DNA extraction.

### DNA extraction

Each fecal specimen was sieved, and the filtrates were concentrated and washed three times with distilled water by centrifugation for 10 min at 1500 *g*. Genomic DNA from fecal samples was extracted using the EZNA^®^ Stool DNA Kit (Omega Bio-tek, Norcross, GA, USA), according to the manufacturer’s recommended procedure. DNA was eluted in 200 μL Solution Buffer from the kit and stored at −20 °C until polymerase chain reaction (PCR) analysis.

### Genotyping of *Cryptosporidium* spp.

A nested PCR targeting a ∼830-bp fragment of the SSU rRNA sequence was used to determine the *Cryptosporidium* species/genotype. The primers were F1 (5′-CCCATTTCCTTCGAAACAGGA-3′) and R1 (5′-TTCTAGAGCTAATACATGCG-3′) for the primary PCR and F2 (5′-AAGGAGTAAGGAACAACCTCCA-3′) and R2 (5′-GGAAGGGTTGTATTATTAGATAAAG-3′) for the secondary PCR [29]. The annealing temperature (°C) was 55 °C and 58 °C for the primary and secondary PCRs, respectively. TaKaRa Taq DNA Polymerase (TaKaRa Bio Inc., Tokyo, Japan) was used for the PCR amplifications. Positive and negative controls were included in each amplification. Secondary PCR products were subjected to electrophoresis in a 1.5% agarose gel and visualized by staining the gel with ethidium bromide.

### Sequence analysis

All secondary PCR products of the expected size (about 830 bp) were directly sequenced at the BioSune Biotechnology Company (Shanghai, China) using an ABI 3730 DNA Analyzer (Applied Biosystems, Foster City, CA, USA). The nucleotide sequences of each obtained in the present study were aligned and analyzed using the Basic Local Alignment Search Tool and Clustal X (http://www.clustal.org/), with reference sequences retrieved from GenBank to identify *Cryptosporidium* species/genotypes [26].

### Phylogenetic analyses

To support the grouping of a genotype, a phylogenetic relationship was assessed using the Phylip package, version 3.69 [24]. Based on the calculated evolutionary distances, neighbor-joining trees were constructed at each locus using the Kimura two-parameter model. Phylograms were drawn using MEGA 6.0 software. The MegAlign program in the DNA Star software package (version 5.0) was used to determine the degree of sequence identity. The unique partial nucleotide sequences of the SSU rRNA were deposited in the GenBank database under accession numbers MF327253, MF327254, and MK135778.

### Statistical analysis

The chi-squared test was used to compare the occurrence of *Cryptosporidium* spp. between different pet markets. Differences were considered statistically significant when *p* < 0.05.

## Results

### Occurrence of *Cryptosporidium* spp.

The overall occurrence of *Cryptosporidium* spp. in pet red-bellied tree squirrels was 1.4% (4/287; 95% CI [0.03–2.75]). The occurrences in pet markets no. 1–4 were 3.4% (2/59), 1.22% (1/85), 0% (0/64), and 1.27% (1/79), respectively, suggesting that the presence of *Cryptosporidium* spp. is generally uncommon (Table 1). The occurrence of *Cryptosporidium* spp. between the four markets were not significant (*χ*^2^ = 2.654, *df* = 3, *p* > 0.05).

**Table 1:** Occurrence and species/genotypes of *Cryptosporidium* in pet red-bellied tree squirrels from different sources in Southwestern China.

Source	No. of examined	No. of positive	Occurrence (%)	95% confidence intervals	Species/genotypes (*n*)
Pet market 1	59	2	3.40	−0.012–0.080	*Cryptosporidium* rat genotype II (2)
Pet market 2	85	1	1.22	−0.011–0.035	*C. wrairi* (1)
Pet market 3	64	0	0		
Pet market 4	79	1	1.27	−0.012–0.037	*C. parvum* (1)
Total	287	4	1.39	0.0003–0.028	*Cryptosporidium* rat genotype II (2), *C. wrairi* (1), *C. parvum* (1)

### *Cryptosporidium* species/genotypes

**Figure 1:**
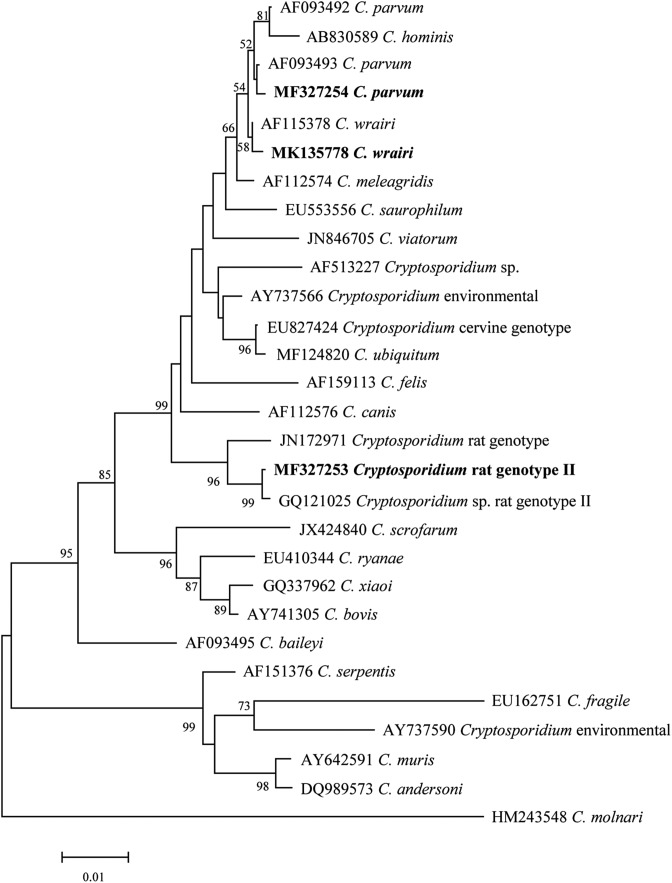
Phylogenetic relationship of *Cryptosporidium* red-bellied tree squirrel isolates in this study to other known *Cryptosporidium* species inferred by neighbor-joining analysis of the 18S rRNA gene based on evolutionary distances calculated using the Kimura 2-parameter model. Bootstrap values were obtained using 1000 pseudo-replicates, with only values above 50% reported.

Four *Cryptosporidium*-positive samples were successfully sequenced, and three *Cryptosporidium* species/genotypes were identified based on sequence analysis of the SSU rRNA gene (Fig. 1). The nucleotide sequences of no. 30 and no. 32 are the same, and the two fecal samples were taken from two red-bellied tree squirrels in pet market 1. In the SSU rRNA gene, there were 1–4 nucleotide differences between the red-bellied tree squirrel isolates and other known *Cryptosporidium* rat genotypes and *C. parvum* isolates. Nucleotide sequence comparison revealed genetic diversity between the two sequences from isolates 30 and 44. Isolate 30 contained four single-nucleotide polymorphisms (SNPs) within the 288–289 bp and 464–465 bp regions of the SSU rRNA gene sequence of *Cryptosporidium* (deletions: A/A, T/C), whereas isolate 44 contained four SNPs within the 37 bp (transversions: A/T), 38–39 bp (deletion: TA), and 702 bp (addition: T) regions compared to the rat *Cryptosporidium* variant JX294365, with which it shared 99.36% homology. Isolate 44 had 99.86% homology with the partial sequence of the 18S ribosomal RNA gene of the *C. parvum* isolate KU198182. Isolate 60 shared 99.63% homology to the guinea pig *C. wrairi* isolate from Atlanta, USA (U11440).

## Discussion

*Cryptosporidium* spp. have been reported in rodents from nine different countries, including developed countries (USA, Australia, Japan, Slovak Republic, and Italy) and developing countries (Philippines, Iran, Nigeria, and China) [12, 22, 23]. Additionally, *Cryptosporidium* spp. has been detected in various rodent species. In this study, the occurrence of *Cryptosporidium* spp. was 1.4%, which is consistent with previous reports on the laboratory rat in Nigeria (2/134, 1.5%) [1], laboratory mouse (4/229, 1.7%) and bamboo rat (3/92, 3.3%) in China [17], and brown rat in Iran (6/91, 6.6%) [19]. However, the occurrence in this study was lower than that in pet chinchillas in China (10%, 14/140), the Asian house rat in the Philippines (44.6%, 37/83), striped field mouse in the Slovak Republic (33%, 34/103), and red squirrel in Italy (24.3%, 17/70) [16, 20, 23]. These differences may be explained by many factors, such as the number of samples, different geography of the source region, host health status, and raising management system. In this study, a relative lower occurrence of *Cryptosporidium* spp. may be due to the fact that red-bellied tree squirrels were kept in a separate cage and have a lower feeding density, which contribute to reducing the transmission possibility between infected squirrels and other squirrels.

Currently, five *Cryptosporidium* species (*C. parvum*, *C. muris*, *C. andersoni*, *C. ubiquitum*, and *C. wrairi*) and nine *Cryptosporidium* genotypes (mouse genotype I, rat genotypes II–IV, ferret genotype, suis-like genotype, *C. hominis* monkey genotype II, chipmunk genotype III, and hamster genotype) have been reported in various rodents in China [6, 13, 31]. Among them, *C. parvum* is the most common zoonotic *Cryptosporidium* species and has been found in humans worldwide [10, 29]. It is a zoonotic pathogen and has been reported in humans as well as in many animals in China, such as cattle, dogs, horses, birds, giant pandas, red pandas, deer, snakes, and rodents [15, 27, 30]. *Cryptosporidium* rat genotype II has been found in brown rats in the Philippines [20], Nigeria [1], Australia [21], and China. *C. wrairi* was first detected in rodents in guinea pigs in China [5].

Phylogenetic trees were constructed for the SSU rRNA locus of the four red-bellied tree squirrel isolates (Fig. 1). Similar topologies were produced in neighbor-joining trees for the SSU rRNA locus, and the four isolates (two *Cryptosporidium* rat genotype II, one *C. parvum*, and one *C. wrairi*) referenced from GenBank formed a cluster supported by bootstrap values. Thus, based on our genotyping data, four isolates from this study represent *Cryptosporidium* rat genotype II, one *C. parvum*, and one *C. wrairi.* Despite considerable research on *Cryptosporidium* spp., only a few genetic studies have documented their occurrence in rodents; therefore, red-bellied tree squirrels, similarly to other rodents, may play a role in the transmission of *Cryptosporidium* to humans [14].

In conclusion, this is the first report of *Cryptosporidium* spp. in red-bellied tree squirrels in China. Three species/genotypes were identified: *C. parvum*, *C. wrairi*, and *Cryptosporidium* rat genotype II. Due to *C. parvum* having been reported in humans, our findings suggest that red-bellied tree squirrels may act as potential reservoirs for zoonotic *Cryptosporidium* spp. for transmission to humans. Moreover, due to the widespread practice of keeping pet rodents in China and their high frequency of contact with humans, proper advice should be given to the susceptible human populations in order to reduce the zoonotic transmission of this neglected disease.
